# DMBT1 expression and neutrophil-to-lymphocyte ratio during necrotizing enterocolitis are influenced by impaired perfusion due to cardiac anomalies

**DOI:** 10.1186/s40348-021-00133-9

**Published:** 2022-01-06

**Authors:** Sonja Diez, Manuel Besendörfer, Veronika Weyerer, Arndt Hartmann, Julia Moosmann, Christel Weiss, Marcus Renner, Hanna Müller

**Affiliations:** 1 Pediatric Surgery, Department for General Surgery, University Hospital Erlangen, Friedrich-Alexander-Universität Erlangen-Nürnberg (FAU), Loschgestraße 15, 91054 Erlangen, Germany; 2 Institute of Pathology, Friedrich-Alexander-Universität Erlangen-Nürnberg (FAU), Krankenhausstraße 8-10, 91054 Erlangen, Germany; 3 Department of Pediatric Cardiology, Friedrich-Alexander-Universität Erlangen-Nürnberg (FAU), Loschgestraße 15, 91054 Erlangen, Germany; 4 Department of Medical Statistics & Biomathematics, Medical Faculty Mannheim, Heidelberg University, Theodor-Kutzer-Ufer 1-3, Haus 3, Ebene 4, 68167 Mannheim, Germany; 5 Institute of Pathology, Heidelberg University, Im Neuenheimer Feld 224, 69120 Heidelberg, Germany; 6 Division of Neonatology and Pediatric Intensive Care, Department of Pediatrics, Friedrich-Alexander-Universität Erlangen-Nürnberg (FAU), Loschgestraße 15, 91054 Erlangen, Germany; 7 Neonatology and Pediatric Intensive Care, Department of Pediatrics, University of Marburg, Baldingerstraße, 35033 Marburg, Germany

**Keywords:** DMBT1, Deleted in malignant brain tumors 1, Necrotizing enterocolitis, Congenital heart disease, Neutrophil-to-lymphocyte ratio, Monocyte-to-lymphocyte ratio

## Abstract

**Background:**

Deleted in malignant brain tumors 1 (DMBT1) is involved in innate immunity and epithelial differentiation. It has been proven to play a role in various states of inflammation or hypoxia of fetal gastrointestinal and pulmonary diseases. Discrimination of pathogenesis in necrotizing enterocolitis (NEC) based on cardiac status improves the understanding of NEC in different patient subgroups. We aimed at examining DMBT1 expressions regarding their association with cardiac status leading to impaired intestinal perfusion, intraoperative bacteria proof, and a fulminant course of NEC.

**Methods:**

Twenty-eight patients with NEC were treated surgically between 2010 and 2019 at our institution. DMBT1 expression was examined in intestinal sections using immunohistochemistry to detect DMBT1 protein. Associations of clinical parameters and DMBT1 expression were analyzed.

**Results:**

We examined DMBT1 levels in 10 patients without cardiac defects and 18 patients with persisting ductus arteriosus (PDA) and congenital heart defects (CHD). Compared to patients without cardiac malformations, DMBT1 levels tended to score higher in patients with PDA/CHD (*p* = 0.2113) and were negatively correlated with C-reactive protein in these infants (*p* = 0.0172; *r* = − 0.5533). The number of DMBT1-expressing macrophages was elevated in the PDA/CHD-subgroup (*p* = 0.0399). Ratios of neutrophils and monocytes to lymphocytes were significantly higher in infants with PDA/CHD (*p* = 0.0319 and 0.0493). DMBT1 expression was significantly associated with positive bacterial culture of intraoperative swabs (*p* = 0.0252) and DMBT1 expression of the serosa was associated with a fulminant course of NEC (*p* = 0.0239).

**Conclusions:**

This study demonstrates that DMBT1 expression may be influenced by cardiac anomalies with an impaired intestinal perfusion in the neonatal intestine. NEC in PDA/CHD infants is associated with more DMBT1-positive macrophages and a significantly elevated neutrophil-to-lymphocyte ratio.

## Background

Necrotizing enterocolitis (NEC) is a very serious disease in preterm and term infants. Incidence rates depend on birth weight and prematurity with a maximum of up to 15% of affected neonates born at less than 30 weeks of gestational age or birth weight of < 1500 g [1]. In contrast, almost 10% of all patients diagnosed with NEC are term neonates, 50% of whom are presenting with persistent ductus arteriosus (PDA) or/and congenital heart disease (CHD) [2]. The increased risk for NEC in these cases is caused by the steal phenomenon leading to reduced perfusion of the intestine and—at least in cyanotic CHD and in preterm infants presenting multiple oxygen desaturations due to PDA—by additional hypoxia. NEC and CHD are shown to be interrelated as main factors for morbidity and mortality in neonates. There is an increased risk of mucosal ischemia due to circulatory alterations, provoking intestinal necrosis [3]. Multiple factors are additionally described as risk factors for NEC (e.g., chorioamnionitis, placental insufficiency, gut ischemia) [4]. The immature immune response of infants and particularly in preterm infants hereby substantially contributes to NEC: intestinal innate and adaptive immunity and inflammatory control are immature and lead to dysfunction of the intestinal barrier [5–7]. These risk factors seem to alter the intestinal epithelial barrier, as a result of which bacteria are able to translocate from the intestinal lumen into the sub-epithelial tissue and induce inflammation [4]. NEC thus remains a multifactorial induced disease. Research of the last decade therefore focused on the separation of pathology of NEC in patient subgroups [8–11]. Various publications were able to confirm differences in incidence, mortality, and clinical factors of patients with CHD with concomitant steal phenomenon. In these patients, pathophysiology is primarily based on an impaired intestinal circulation with resulting ischemia and elevated levels of circulating endotoxins and proinflammatory cytokines. A recent publication from Neu proposed to completely separate this intestinal necrosis due to hypoxic mechanisms seen in full-term neonates from the classical NEC of preterm infants [12] as did the most recent study of Klinke et al. [13]. We were recently able to describe that infants with PDA/CHD had more frequently macroscopic intestinal necrosis and positive bacterial culture of intraoperative swabs compared to preterm infants without PDA/CHD [11].

Deleted in malignant brain tumors 1 (DMBT1) is a secreted protein with functions in innate immunity, epithelial cell differentiation, angiogenesis, and tumorigenesis. As member of the secreted scavenger receptor cysteine rich (SRCR) protein family it is involved in various processes of inflammation [14]. This includes binding to various bacteria and interaction with several defense factors (e.g., surfactant protein A/D, secretory IgA) [15, 16]. DMBT1 is expressed in the fetal gastrointestinal tract and additionally in the small and large intestine of preterm infants [17]. The basal and luminal localization in epithelial cells point to functions in epithelial differentiation (basal DMBT1) and local innate immunity (luminal DMBT1). This was supported by the fact that DMBT1 is enormously upregulated in inflammatory processes such as NEC independently from gestational age [17]. The promotor of DMBT1 furthermore contains a binding site for hypoxia-inducible factor 1α, which could be observed with the upregulation of DMBT1 expression in lung tissues and lung epithelial cells after hypoxia [18].

The aim of this study was to compare intestinal DMBT1 expression, intraoperative bacterial detection and the clinical course in infants with NEC according to their cardiac anatomy and to provide new insides into the pathophysiology of different NEC subtypes.

## Patients and methods

### Patients

The study’s cohort of NEC patients was treated throughout the years 2008 to 2019 at the University Hospital Erlangen. Formalin-fixed and paraffin-embedded sections were available after surgical resection of affected intestinal segments. Gestational ages were defined as time elapsed between the first day of the last menstrual period and the day of birth, ranging from 23 to 39 weeks. We documented different clinical factors within the course of NEC (vital parameters, laboratory findings, medication, and recovery), concomitant diseases, and surgical approach. Diagnosis of reduced intestinal perfusion in patients with PDA or CHD was made based on sonography and Doppler. The definition of a relevant PDA included hereby a modified intestinal perfusion, the lack of pressure separation between both circuits and the size increase of the left atrium. Neutrophil-to-lymphocyte ratio (NLR) and monocyte-to-lymphocyte ratio (MLR) were calculated using the following formulas at the time of indication for surgical therapy of NEC: NLR = absolute neutrophil count/absolute lymphocyte count; MLR = absolute monocyte count/absolute lymphocyte count. C-reactive protein was also determined at time of diagnosis. An intraoperative swab was performed during surgery to enable bacterial culture. NEC was diagnosed and staged by modified Bell’s criteria [19]. Decision for a surgical approach was made based on the German AWMF clinical guidelines on NEC including the confirmation of intestinal perforation or the infant’s deterioration during the conservative treatment course [20]. In the latter, velocity of progression into a septic clinical picture led to indication for immediate surgery. In a minor part of very immature preterm infants with severe cardiac malformations, immediate surgery was performed in NEC IIb to enable survival. In these cases, a high possibility of unstoppable intestinal necrosis and irreversible deterioration was predictable without surgery. The following criteria were included in the diagnosis: changes in blood pressure and respiratory parameters, need of fluids to maintain circulation, and need for catecholamines and advanced mechanical ventilation to achieve satisfying oxygen levels. There were no differences in surgical indication between preterm patients and patients with cardiac concomitant diseases, as the same surgical team led diagnostic and therapeutic management in all patients within the whole study time.

Fulminant NEC was retrospectively defined based on clinical experience as there is no consistent definition in literature: definition included NEC with extensive necrosis, requiring multiple surgical interventions or intestinal resection of > 20 cm. The study was approved by the local ethical committee (University of Erlangen-Nürnberg, Germany; No 281_19Bc) and was performed in accordance with the 1964 Helsinki Declaration and its later amendments or comparable ethical standards. Infants were divided into two groups to analyze differences in NEC according to impaired intestinal perfusion due to cardiac abnormalities (see above). The first group comprised infants without cardiac anomalies. The second group included infants with relevant PDA or CHD.

### Immunohistochemistry

Formalin-fixed and paraffin-embedded intestinal sections of 28 neonates (10 infants without cardiac anomalies and 18 infants with PDA/CHD) were analyzed by immunohistochemistry to detect DMBT1 (one section for each patient). The sections were stained by the Tissue Bank of the National Center for Tumor Diseases Heidelberg, Germany, in accordance with the regulations of the tissue bank. Immunohistochemistry was performed using the rabbit polyclonal antiserum anti-DMBT1p84 as previously described [16, 17, 21–23]. Each tissue section was analyzed with a magnitude of 1:40, 1:100, and 1:200. A score for determining the intensity and extent of DMBT1 signals was established. DMBT1-positive tissues were scored semi-quantitatively from 0 (no staining), 1 (weak staining), 2 (moderate staining) to 3 (highly intense staining). Additionally, we semi-quantitatively analyzed the number of DMBT1-positive macrophages: 0 (no DMBT1-positive macrophages), 1 (few DMBT1-positive macrophages), 2 (moderate number of DMBT1-positive macrophages), and 3 (many DMBT1-positive macrophages). An average score of the parts with high inflammation and/or perforation was determined for each infant, combining the results of all inflammatory regions. Each section was scored independently by two blinded investigators (SD, HM) and the average score was used for statistics. A score of total DMBT1 expression as well as separate DMBT1 scores of macrophages, endothelia, and serosa were determined.

### Statistical analysis

Quantitative variables and DMBT1 score are presented as median together with minimum and maximum values. For qualitative factors, absolute and relative frequencies are given. In order to compare two groups, exact Mann-Whitney *U* test or Fisher’s exact test has been used, as appropriate. Each 2-group comparison was conducted as a 2-sided test. Correlation coefficients according to Pearson have been assessed to quantify the strength of correlation between two quantitative variables.

Test results with *p* values smaller than 0.05 were regarded as statistically significant. SAS software, release 9.4 (SAS Institute Inc., Cary, NC, USA), was used for all statistical calculations.

## Results

### Baseline clinical variables of patients

Within the study period (2008–2019), 97 neonates were treated with NEC in our perinatal center. Of these, 19 patients (20%) were confirmed with a very low birth weight of < 1000 g (VLBW) and 36 patients (37%) were diagnosed with concomitant CHD or PDA. Thirty-six of these 97 patients (37%) underwent surgery due to diagnosis of progressed intestinal inflammation and are focused on this study. In 28 of these patients, parts of the intestine were resected. Gestational age at birth of the 28 infants ranged between 23.6 and 39.6 weeks (median 32.7 weeks). Diagnosis of SGA (small for gestational age) was made in 7 patients (25%). Surgical intervention was performed between day 3 and 74 of life (median 19 days), corresponding to gestational ages of 29.6 to 39.6 weeks (median 35.5 weeks). In 57% of cases, sonographic or radiological signs of an intestinal perforation could be observed. In the remaining patients (43%), immediate surgery was conducted in cases of a high possibility of unstoppable intestinal necrosis and irreversible deterioration without surgery. Two groups of infants were determined according to cardiac anatomy: The first group contained 10 infants without cardiac anomalies, the second group consisted of 18 infants, 5 patients of which presented with PDA and 13 infants with CHD, both resulting in impaired intestinal perfusion and—at least in a part of theses infants—in an impaired oxygenation due to recurrent oxygen desaturations or continuous reduced oxygen saturation. In all of these patients, an impact on intestinal diastolic flow could be seen in Doppler sonography (reduced or even reversed end-diastolic flow). Basic demographic and clinical data of both groups are detailed in Table 1.

**Table 1:** Basic demographic and clinical data of the study population. Significant values are highlighted

Clinical parameter	Whole population (*n* = 28, 100%)	Preterm patients without cardiac disease (*n* = 10)	Patients with cardiac disease (*n* = 18)	*p* value
Sex [*n* (%)]				
Male Female	17 (71%) 11 (39%)	6 (60%) 4 (40%)	11 (61%) 7 (39%)	1.000
Gestational age [weeks [median (range)]]	32.7 (23.6–39.6)	31.1 (25.3–35.6)	36.5 (23.6–39.6)	0.142
Weight at birth [g [median (range)]]	1940 (550–3300)	1670 (780–2160)	2205 (550–3300)	0.245
Leading cardiac malformations [*n* (%)] Persisting ductus arteriosus Atrial/ventricular septal defect Pulmonary stenosis with double outlet ventricle Transposition of the great arteries Hypoplastic left heart syndrome Tetralogy of Fallot Ebstein’s anomaly	18 (64%) 5 (18%) 2 (7%) 2 (7%) 3 (11%) 3 (11%) 2 (7%) 1 (3%)	0	18 (100%) 5 (28%) 2 (11%) 2 (11%) 3 (17%) 3 (17%) 2 (11%) 1 (6%)	**< 0.0001** 0.128 0.524 0.524 0.533 0.533 0.524 1.000
BELL-stage [*n* (%)]				
IIb IIIa IIIb	10 (36%) 2 (7%) 16 (57%)	4 (40%) 1 (10%) 5 (50%)	6 (33%) 1 (6%) 11 (61%)	0.854
Perforation [*n* (%)]				
Yes No	16 (57%) 12 (43%)	5 (50%) 5 (50%)	11 (61%) 7 (39%)	0.698
Localization of NEC [*n* (%)]				
Small bowels Colon Both localizations	10 (36%) 11 (39%) 7 (25%)	5 (50%) 3 (30%) 2 (20%)	5 (28%) 8 (44%) 5 (28%)	0.602
Proof of bacteria (intraperitoneal swabs) [*n* (%)]				
Yes No	12 (43%) 16 (57%)	1 (10%) 9 (90%)	11 (61%) 7 (39%)	**0.016**
Proof of bacteria (intraperitoneal swabs) [*n* (%), multiple answers possible/patient]				
*E. coli* *Enterobacter cloacae* *Enterococcus* *Staphylococcus* *Serratia marcescens* *Citrobacter koseri*	3 (11%) 1 (4%) 4 (14%) 7 (25%) 1 (4%) 1 (4%)	1 (10%) 1 (10%)	3 (17%) 1 (6%) 3 (17%) 6 (33%) 1 (6%) 1 (6%)	0.533 1.000 1.000 0.364 1.000 1.000
Fulminant NEC [*n* (%)]				
Yes No	8 (29%) 20 (71%)	2 (20%) 8 (80%)	6 (33%) 12 (67%)	0.669
Short bowel syndrome [*n* (%)]				
Yes No	5 (18%) 23 (82%)	2 (20%) 8 (80%)	3 (17%) 15 (83%)	1.000
Survival [*n* (%)]				
Yes No	20 (71%) 8 (29%)	9 (90%) 1 (10%)	11 (61%) 7 (39%)	0.194
pH at diagnosis [median (range)]	7.32 (7.04–7.53)	7.31 (7.10–7.53)	7.33 (7.04–7.50)	0.488
Base excess at diagnosis [mmol/l [median (range)]]	− 4.3 (− 17.0 to 6.3)	− 6.9 (− 17.0 to − 2.1)	− 2.1 (− 9.3 to 6.3)	**0.013**
Lactate at diagnosis [mmol/l [median (range)]]	2.5 (1.0–11.4)	2.3 (1.4–8.8)	3.0 (1.0–11.4)	0.924
White blood cell count at diagnosis [*n* × 10^9^/l [median (range)]]	4.5 (0.5–23.1)	4.5 (0.5–9.3)	4.5 (1.3–23.1)	0.796
Platelet count at diagnosis [*n*/μl [median (range)]]	193.0 (22.0–571.0)	214.0 (22.0–314.0)	188.0 (51.0–571.0)	0.832
CrP at diagnosis [mg/l [median (range)]]	40.9 (0.6–259.0)	10.2 (0.6–259.0)	52.7 (10.8–220.3)	0.160

Includes basic demographic and clinical data of the 28 included patients

### NLR and MLR in dependence of cardiac anatomy

**Fig. 1:**
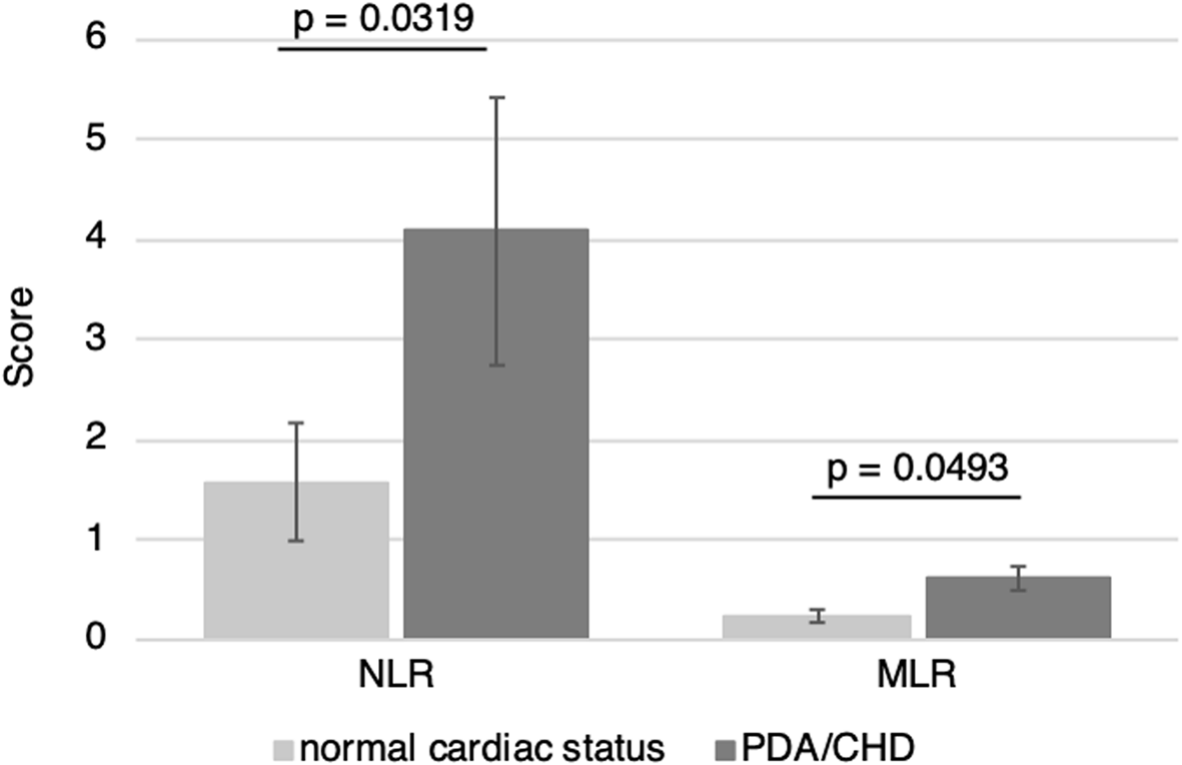
Neutrophil-to-lymphocyte ratio (NLR) and monocyte-to-lymphocyte ratio (MLR). NLR and MLR were significantly higher in infants with persistent ductus arteriosus (PDA)/congenital heart disease (CHD) in comparison to infants with normal cardiac anatomy

Both ratios (NLR and MLR) were significantly higher in infants with PDA/CHD in comparison to infants with normal cardiac anatomy (*p* = 0.0319 and 0.0493, respectively; see Fig. 1 and Table 2), even though there was no significant difference in C-reactive protein levels between both groups (*p* = 0.1599 two-tailed test; Table 1).

**Table 2:** Neutrophil-to-lymphocyte ratio (NLR) and monocyte-to-lymphocyte ratio (MLR) distribution within the study population. Significant values are highlighted

Clinical parameter	Whole population (*n* = 28, 100%)	Preterm patients without cardiac disease (*n* = 10)	Patients with cardiac disease (*n* = 18)	*p* value
NLR [median (range)]	1.7220 (0.125–25.000)	1.0393 (0.300–6.700)	2.1976 (0.125–25.000)	**0.032**
MLR [median (range)]	0.2917 (0–2.000)	0.2289 (0–0.667)	0.3828 (0.063–2.000)	**0.049**

### Total DMBT1 expression of inflamed intestine during NEC

**Fig. 2:**
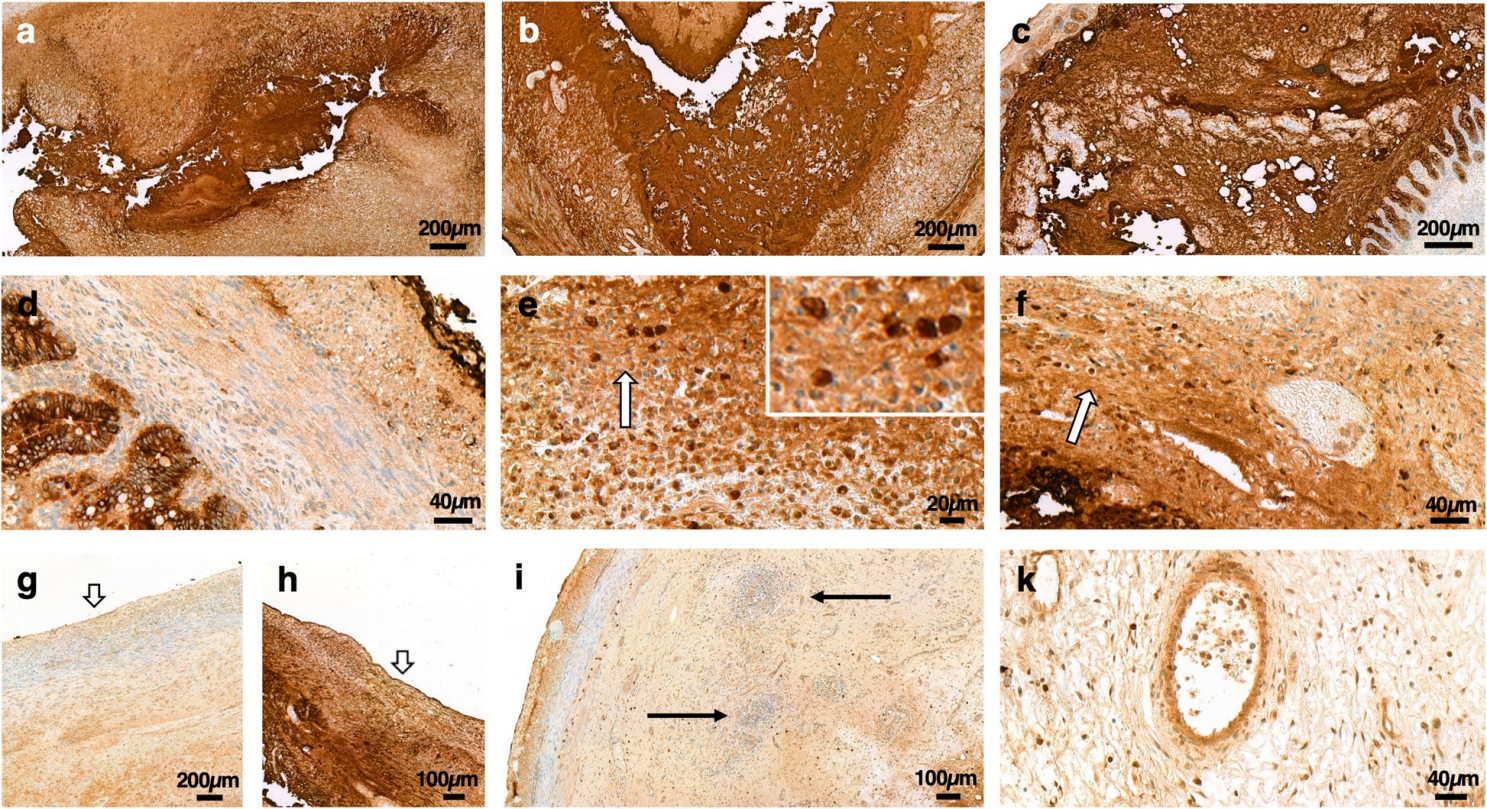
DMBT1 expression in infants with necrotizing enterocolitis (NEC) depending on cardiac status. Representative illustration of high DMBT1-expression levels in NEC infants with intraoperative bacteria detection with normal cardiac status (**a**) and with CHD/PDA (**b** and **c**, respectively). **d**–**f** Infants with PDA/CHD (**e** and **f**, respectively) show significantly more DMBT1-positive macrophages (arrows) in comparison to preterm infants with normal cardiac status (**d**). Insert in **e**: higher magnification of DMBT1-positive macrophages. **g/h** DMBT1-expression of serosa (arrows) was higher in NEC infants with fulminant course (**h**) in comparison to infants with non-fulminant course (**g**). **i** The invasion of leukocytes into the intestinal wall (arrows) in an infant with CHD and NEC. **k** DMBT1 expression in endothelial cells in some neutrophils

Compared to infants with normal cardiac anatomy, infants with PDA/CHD tended to show higher total DMBT1 score without reaching statistical significance (median score: 2.71 versus 2.00; *p* = 0.2113). The total DMBT1 score was negatively correlated with C-reactive protein in infants with PDA/CHD (*p* = 0.0172; *r* = − 0.5533), which was not observed in the group without cardiac anomalies (*p* = 0.4357; *r* = 0.2786). DMBT1 score was significantly associated with positive bacterial culture of intraoperative swabs (*p* = 0.0252), in which staphylococcus and streptococcus species as well as a variety of stool bacteria were detectable: the median total DMBT1 score in infants without bacterial proof was 2.00 and with proof of bacteria 3.00 (Fig. 2a–c). However, a fulminant NEC course was not significantly correlated with total DMBT1 scores (*p* = 0.163), even though the median total DMBT1 score was slightly higher in comparison to infants without fulminant NEC (3.00 versus 2.25). This difference was even more highlighted when excluding preterm infants with cardiac malformations from the analysis (median scores: 3.0 versus. 2.00), although it slightly failed to reach statistical significance (*p* = 0.0830).

### DMBT1 expression of intestinal macrophages during NEC

We analyzed the number of DMBT1-expressing macrophages. DMBT1 expression in submucosal macrophages in infants with PDA/CHD anomalies showed significantly more DMBT1-positive macrophages in comparison to infants without PDA or cardiac malformations (median DMBT1 score of macrophages: 2.00 versus 1.38; *p* = 0.0399; Fig. 2d–f). To ensure statistical accuracy without influences of prematurity of 10 infants with cardiac malformations, we performed another analysis, excluding these 10 newborns. Event then, this difference remained significant (*p* = 0.0459). DMBT1 scores in macrophages showed no association with a fulminant course (median DMBT1 score of macrophages: 1.88 in patients with a fulminant course versus 1.71 in patients with non-fulminant NEC; *p* = 0.4438).

### DMBT1 expression of intestinal endothelia during NEC

**Fig. 3:**
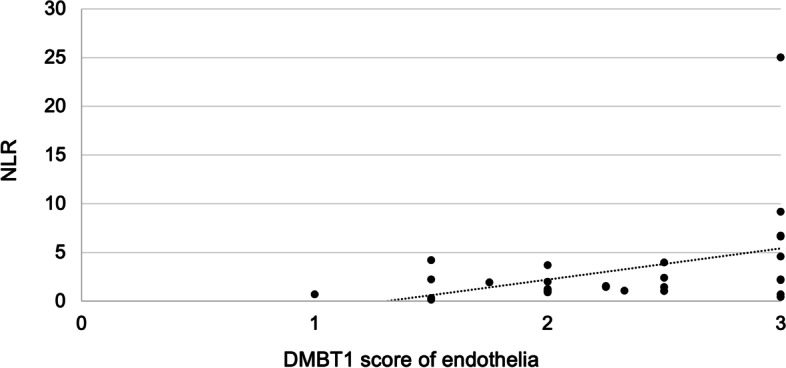
DMBT1 expression in association with neutrophil-to-lymphocyte ratio (NLR). A significant correlation of DMBT1 expression (scored semi-quantitatively from 0 (no staining), 1 (weak staining), 2 (moderate staining) to 3 (highly intense staining) with NLR could be confirmed (*p* = 0.0352; *r* = 0.3994)

We analyzed DMBT1 expression of endothelial cells in the affected intestine of infants with NEC (Fig. 2k). DMBT1 expression of intestinal endothelial cells was significantly associated with NLR (*p* = 0.0352; *r* = 0.3994; Fig. 3) but not with MLR (*p* = 0.1467). However, there were no significant associations between DMBT1 expression and cardiac anomalies (*p* = 0.5384), a positive bacterial culture of intraoperative swabs (*p* = 0.5652) or a fulminant NEC course (*p* = 0.1658). Furthermore, invasion of leukocytes, mainly neutrophils, into the intestinal wall was observed (Fig. 2i). Some neutrophils showed DMBT1-expression (Fig. 2k).

### DMBT1 expression of intestinal serosa during NEC

Infants with fulminant NEC showed a higher DMBT1 expression score of the serosa in comparison to infants without fulminant NEC course (median DMBT1 score of serosa: 2.88 versus 2.00). This difference slightly failed to reach statistical significance (*p* = 0.0239; Fig. 2g/h). Positive bacterial culture of intraoperative swabs (*p* = 0.5172) or cardiac status (*p* = 0.2467) did not correlate with DMBT1 expression of the serosa.

## Discussion

Different studies support the critical role of DMBT1 in epithelial differentiation, fetal, and neonatal innate immunity [16, 17] and especially functions of DMBT1 in the intestine [23]. Accordingly, increased DMBT1 expression with increasing gestational ages has been previously demonstrated in the small intestine of human fetus (gestational age of 14–35 weeks) and in neonates. An upregulation of DMBT1 has been proven in neonatal gastrointestinal diseases to be associated with inflammation (e.g., necrotizing enterocolitis) and infection in preterm patients (e.g., severe sepsis) independent from gestational age [17]. In this study, we focused on NEC in preterm and term neonates with different cardiac anatomy to analyze potential new pathophysiological mechanisms of different NEC subgroups. We postulate that DMBT1 might be differentially expressed regarding the immaturity of the innate immunity alone versus in combination with impaired intestinal perfusion and consecutive cellular hypoxia, since inflammation and hypoxia are known factors which influence DMBT1 expression [17, 18]. Elevated circulating cytokines are described as part of the systemic inflammatory response during NEC, which may also play a direct or indirect role in augmenting the mucosal injury [4].

DMBT1 acts as a binding protein to multiple bacteria, such as group A and B *Streptococci*, *Staphylococcus aureus*, and *E. coli* [16]. The binding results in suppression of bacterial invasion and hindrance of bacterial infections [24]. NEC develops in response to an abnormal interaction of exaggerated bacterial signaling in the premature intestine [4], in which *E. coli* plays a major role. The results of our study confirm an association of positive intraabdominal swabs and the presence of CHD/PDA, as demonstrated in a recent study [11] and show a predominance of *E. coli* and *Staphylococcus* in intraabdominal swabs. DMBT1 addresses a critical factor in the pathogenesis of NEC. Upregulation of DMBT1 in response to bacterial invasion is supported by our observation that the total DMBT1 score was significantly associated with positive bacterial culture of intraoperative swabs. However, an association between different bacterial species and DMBT1 levels could not be confirmed. DMBT1 is able to bind various species of bacteria. Higher levels of DMBT1 can bind a higher load of bacteria [24, 25]. The elevated total DMBT1 expression of the serosa in fulminant NEC also emphasized the positive relation between intense and pronounced inflammation of the intestinal wall and DMBT1 expression.

The total intestinal DMBT1 expression level was slightly but not significantly higher in infants with PDA/CHD compared to infants without cardiac anomalies. This corresponds to the higher inflammation and supports the results of our own data indicating that infants with PDA/CHD had more frequently macroscopic intestinal necrosis and positive bacterial culture of intraoperative swabs compared to preterm infants without PDA/CHD [11]. Cellular hypoxia may also lead to higher DMBT1 expression in intestinal epithelium. This observation has already been made in the respiratory epithelium [18]. Even though the gestational ages between both groups (preterm infants versus infants with PDA/CHD) were slightly different (31.1 versus 36.5 weeks), this cannot explain the differences in DMBT1 expression as shown in our previous study, as upregulation of DMBT1 is independent from gestational age and only the basal level of intestinal DMBT1 expression depends on immaturity [17].

The reduced perfusion and reduced nutrition of the intestine in infants with PDA/CHD may represent an explanation for the negative correlation between DMBT1 levels and CRP. Even cases with the highest DMBT1 expression did not show CRP values > 50 mg/l in every case. This demonstrates that there is a very intense local inflammation with high induction of DMBT1 expression, but the systemic reaction with CRP production is often less intense compared to local DMBT1 expression in infants with impaired intestinal perfusion and impaired systemic circulation due to cardiac anomalies. The initiation of systemic CRP requires intestinal perfusion to induce the systemic reaction.

Intestinal macrophages appear at 11–12 weeks of gestational age in human fetal development followed by a rapid increase of the resident macrophage population during the 12–22-week period [26–28]. Intestinal macrophages represent an important host defense mechanism. As the first phagocytic cells of the innate immune system, they encounter luminal bacteria that translocate the intestinal epithelium into the lamina propria. The inflammatory responses of the mucosal macrophages contribute to the risk of mucosal injury during NEC [4]. A macrophage-rich infiltrate is involved in the cellular inflammatory response in NEC [29]. It was shown that intestinal macrophages are normally maintained through continuous recruitment of circulating monocytes followed by in situ differentiation in the lamina propria [30, 31]. The higher amount of DMBT1-positive intestinal macrophages in infants with PDA/CHD in comparison to NEC patients with sufficient intestinal perfusion demonstrated a higher inflammatory response of macrophages in the first mentioned NEC subgroup. Even if our results are only able to represent the absolute numbers of DMBT1-positive macrophages and fail at presenting relative numbers of DMBT-1 positive macrophages, this information might contribute to the extended macroscopic intestinal necrosis and positive bacterial culture of intraoperative swabs observed in infants with PDA/CHD [11].

We further analyzed the NLR and MLR in dependence of cardiac anatomy. Both NLR and MLR are discussed as outcome parameter in adult intensive care unit patients [32]. A rise in blood neutrophils is part of an appropriate inflammatory response [4]. NLR is a simple parameter for easy assessment of a patient’s inflammatory status [33]. Yang et al. reported that the NLR may be a useful marker for early NEC diagnosis and could allow to distinguish the severity of NEC [34]. During NEC, neutrophils emigrate into the intestines and peritoneum, and increased margination of neutrophils in the microvasculature is known [4], which was also seen in our study population (Fig. 2i/k).

Intestinal macrophage populations are recruited from circulating monocytes followed by in situ differentiation in the lamina propria [30, 31]. Accordingly, monocytes guarantee that macrophage-rich infiltrates are present in NEC [29]. We observed higher MLR in infants with PDA/CHD compared to infants with normal cardiac anatomy, supporting the role of monocytes/macrophages in NEC of infants with PDA/CHD.

There are different aspects due to inflammation in NEC. First, the limited mucosal immune system increases the risk of inflammatory injury and NEC in preterm infants. Second, elevated circulating cytokine levels due to intestinal inflammation and increased inflammatory status increase intestinal injury [4]. There is an underlying elevation of circulating endotoxins and proinflammatory cytokines in term and predominantly preterm neonates, which predisposes neonates to intestinal necrosis [6]. The stimulation of nuclear factor κB (NF-κB) is induced by pro-inflammatory stimuli, which subsequently induces the transcription of pro-inflammatory mediators such as interleukin 6 and 8 (IL-6/IL-8), tumor necrosis factor alpha (TNFα), interferon γ, and platelet-activating factor (PAF), ultimately leading to intestinal necrosis. The role of IL-6 in neonatal sepsis or intestinal necrosis has additionally been well established, presenting itself as a promising new biomarker [35]. A correlation of DMBT1-/- mice and colitis could be confirmed, in which elevated levels for IL-6 and TNF during inflammation could be seen [23].

It remains unclear if the increased DMBT1 levels in NEC patients may augment the intestinal injury or may help to bind bacteria [25] and to suppress inflammation as already observed in gut and lung epithelial cells [23, 36]. It may also be possible that DMBT1 has different functions in different time windows.

The study has some limitations: First, both subgroups include preterm infants. To exactly define the exclusive role of gestational age and cardiac malformation on DMBT1 expression as well on NEC manifestation, it may be useful to examine greater study populations and create subgroups enabling examination of the distinct role of prematurity and cardiac malformation. Second, improvement of the pathologic handling of resected intestinal tissue (e.g., longitudinal versus transverse cutting) may enable accurate comparability of tissue samples in terms of assessment of DMBT1-positive structures and in terms of the ratio of DMBT1-positive macrophages.

## Conclusions

In conclusion, we confirm that DMBT1 expression may be influenced by various states of inflammation or cellular hypoxia in the neonatal intestine. The hypothesis of different pathologic conditions in various NEC subtypes is supported by the observation that DMBT1 expression depends on NEC subgroup and that NEC in infants with PDA/CHD is characterized by a significantly increased number of DMBT1-positive macrophages. Furthermore, the neutrophil-to-lymphocyte ratio is significantly higher in infants with an impaired intestinal perfusion due to cardiac anomalies.

## Data Availability

The datasets used and/or analyzed during the current study are available from the corresponding author on reasonable request.
